# Fully automated treatment planning of spinal metastases – A comparison to manual planning of Volumetric Modulated Arc Therapy for conventionally fractionated irradiation

**DOI:** 10.1186/s13014-017-0767-2

**Published:** 2017-01-31

**Authors:** Daniel Buergy, Abdul Wahab M. Sharfo, Ben J. M. Heijmen, Peter W. J. Voet, Sebastiaan Breedveld, Frederik Wenz, Frank Lohr, Florian Stieler

**Affiliations:** 1 Department of Radiation Oncology, Universitätsmedizin Mannheim, Medical Faculty Mannheim, Heidelberg University, Mannheim, Germany; 2 Department of Radiation Oncology, Erasmus MC-Cancer Institute, Rotterdam, The Netherlands; 3 Elekta B.V., De Maas 26, 5684 PL Best, The Netherlands

**Keywords:** Erasmus-iCycle, Automated knowledge-based planning, IMRT, VMAT

## Abstract

**Background:**

Planning for Volumetric Modulated Arc Therapy (VMAT) may be time consuming and its use is limited by available staff resources. Automated multicriterial treatment planning can eliminate this bottleneck. We compared automatically created (auto) VMAT plans generated by Erasmus-iCycle to manually created VMAT plans for treatment of spinal metastases.

**Methods:**

Forty-two targets in 32 patients were analyzed. Lungs and kidneys were defined as organs at risk (OARs). Twenty-two patients received radiotherapy on kidney levels, 17 on lung levels, and 3 on both levels.

**Results:**

All Erasmus-iCycle plans were clinically acceptable. When compared to manual plans, planning target volume (PTV) coverage of auto plans was significantly better. The Homogeneity Index did not differ significantly between the groups. Mean dose to OARs was lower in auto plans concerning both kidneys and the left lung. One hotspot (>110% of D_50%_) occurred in the spinal cord of one auto plan (33.2 Gy, D_50%_: 30 Gy). Treatment time was 7% longer in auto plans.

**Conclusions:**

Erasmus-iCycle plans showed better target coverage and sparing of OARs at the expense of minimally longer treatment times (for which no constraint was set).

**Electronic supplementary material:**

The online version of this article (doi:10.1186/s13014-017-0767-2) contains supplementary material, which is available to authorized users.

## Background

Fractionated or single-dose radiotherapy to spinal metastases of solid tumors is one of the most frequently performed radiation treatments [1]. Highly conformal radiation techniques such as Stereotactic Body Radiation Therapy (SBRT), Intensity Modulated Radiation Therapy (IMRT) or VMAT are increasingly being used as their application becomes easier and dose distributions confer theoretical advantages [2], albeit so far without proof of superior clinical outcome. On the other hand, generation of an optimal IMRT or VMAT plan is often time consuming and it has been shown that staff limitations are correlated with restricted use of new techniques such as IMRT even in the developed world [3, 4]. Automated planning of IMRT and VMAT may reduce the workload which is associated with manual “trial-and-error” approaches by around 50% [5].

Erasmus-iCycle, developed at the Erasmus MC-Cancer Institute, is an optimizer for multicriterial beam profile optimization and beam angle selection for coplanar and non-coplanar IMRT [5–10]. Other solutions for multicriterial beam angle optimization have been proposed by Schreibmann et al. [11], Craft and Monz presented a full multibeam space Pareto navigation tool [12]. However, both are *a posteriori* methods, i.e., the algorithm generates different sets of beam angles and intensity profiles from which the user selects the plan afterward. The Erasmus-iCycle solution is an a priori approach which enables the user to define a site specific set of criteria (wish-list) which may not be violated (constraints) or have to be met as well as possible, or better (objectives). Objectives have assigned priorities to steer the multicriterial planning towards favourable trade-offs between the various treatment goals. If the priority of an objective is higher, the probability that the corresponding objective is met increases. Hard constraints are always respected in Erasmus-iCycle plans.

Beam directions are selected from candidate directions which can be restricted, e.g., in case of coplanar treatments [6, 7, 13]. For fully automated generation of plans that are clinically delivered, Erasmus-iCycle auto plans are automatically reconstructed and segmented in the clinical treatment planning system (TPS) [13]. This study intends to validate VMAT plans for treatment of spinal metastases, generated with this approach, and to compare the quality of automatically generated plans with plans that were manually created by experienced treatment planners. Different spine regions pose different optimization problems as a consequence of different OARs being relevant for the treatment. Therefore cervical, thoracic, and lumbar targets were included in the study design.

## Methods

### Manual plan generation

Forty-two clinical target volumes (CTVs) in the spinal column of 32 patients were manually delineated for clinical routine treatments. CTVs were anisotropically expanded to PTVs that were the basis for all further analyses. All PTVs were reviewed by expert radiation oncologists (FW, FL).

Manual plans were created by expert treatment planners and also reviewed by radiation oncologists. All manually generated plans were calculated using the Monaco® treatment planning system (Elekta Ltd, Crawly, UK) version 3.2 or later which supports static IMRT, dynamic IMRT, and VMAT. Informed consent was obtained from all patients for anonymized processing of their clinical data. The study was approved by the ethics committee of Heidelberg University, Medical Faculty Mannheim (2016-806R-MA). For this study we assumed lungs or kidneys to be (potentially pre-irradiated) OARs, therefore we included only spinal regions which were at the level of kidneys (*n* = 22), lungs (*n* = 17), or both (*n* = 3), resulting in 90 (45*2) OARs and 42 PTVs in total. Spinal reirradiation plans were not tested in this study; nevertheless, we contoured the spinal cord to identify any hotspots in this area within the target volume. Most plans were at the thoracic, thoracolumbar or lumbar level. In 4 cases, soft tissue metastases or rib metastases were included in the PTV. Prescribed doses ranged from 30 to 40 Gy in 10–20 fractions which are commonly applied treatment regimens in patients with sufficient life expectancy and good general condition (Eastern Cooperative Oncology Group, ECOG 0/1; Karnofsky Performance Status, KPS > 80%). Generally, 40 Gy regimens were applied to patients who received postoperative radiotherapy. We did not include patients who had to be treated with spinal cord sparing plans after prior full dose irradiation of the spinal cord. While some patients had in fact received prior radiotherapy, this previous therapy did not require sparing of spinal cord to meet dose constraints [14]. Details on irradiation sites are summarized in Table 1. Further information such as primary tumor site of each patient is provided in Additional file 1: Table S1.

**Table 1:** Characteristics of spinal irradiation plans (*n* = 42)

		Number of sites
Irradiation site	Cervical spine	None
	Cervical and thoracic spine	2
	Thoracic spine^a^	15
	Thoracic and lumbar spine	17
	Thoracic and lumbar spine, Sacrum	3
	Lumbar spine	4
	Lumbar spine and Sacrum	1
Primary tumor site	Breast Cancer	15
	Prostate Cancer	11
	Lung Cancer	5
	Non-Small Cell Lung Cancer	3
	Small Cell Lung Cancer	2
	Gastric and Oesophageal Cancer	5
	Multiple Myeloma	3
	Head and Neck	1
	Unknown Primary	1
	Urothelial Cell Carcinoma	1
Organs at risk	Kidneys	22
	Lungs	17
	Lungs and kidneys in one plan^b^	3
Median dose	40Gy	9
	30Gy	33

Concerning PTV coverage, keeping the patients in both groups would have weighted these 3 plans double, therefore all statistics were recalculated under inclusion/exclusion of these patients. This did not change statistical significance in any case (i.e., only numerical changes occurred)

^a^including 3 plans in which a rib metastasis was included into the irradiation field. In one plan a rib metastasis and a soft tissue metastatic site were included into the irradiation field

^b^in 3 plans, lungs and kidneys were both considered OARs. For statistical considerations, these plans were evaluated in both groups concerning calculations for organs at risk

### Automated VMAT plan generation with Erasmus-iCycle (auto)

Target volumes were identical to those used in the manual planning approach. General principles of iCycle plan generation are described above. Further details on auto-planning with the Erasmus-iCycle/Monaco system have been previously provided by Voet et al. [5]. A site-specific wish-list for our patient cohort is shown in Additional file 2: Table S2. Apart from objectives for the PTV, the kidneys and the lungs, shells around the PTV are used to steer on conformality. Two cases were included which required beam restrictions, both because patients were unable to lift their arms.

### Plan quality and statistical analysis

Plan quality was estimated by calculating the dose to 98% (D_98%_) and to 2% (D_2%_) of the PTV. Both parameters were used to compute the homogeneity index (HI, [15]) which was defined as the ratio between the difference of D_2%_ and D_98%_ and the median dose (D_50%_), i.e.,:

$$ HI=\left[\left({D}_{2\%}-{D}_{98\%}\right)/{D}_{50\%}\right] $$

In addition, we calculated the volume receiving 95% of the prescribed dose (V_95%_ i.e., V_28.5Gy_, or V_38Gy_, depending if the prescribed dose was 30 Gy or 40 Gy), and based on V_95%_ a conformity index (CI, reviewed in [15]) was calculated as follows:

$$ CI={V}_{95\%}/PTV $$

An optimal CI would be considered to be 1; however, values can be above or below 1. To consider both over- and underdosage for statistical comparison of CI, we calculated the difference to 1 as follows:

$$ C{I}_{diff}=\left|CI\hbox{--} 1\right| $$

For OARs, we calculated the mean dose (D_mean_), and in case of the spinal cord, the maximum dose to any hotspot (D_max_). All parameters were compared directly between manually generated plans and auto plans. Differences between variables were computed using the two-sample paired Wilcoxon test. Statistical significance was defined as p ≤ 0.05 (two-sided testing). Clinical acceptability of the plans was evaluated by an expert radiation oncologist (FL).

## Results

**Fig. 1:**
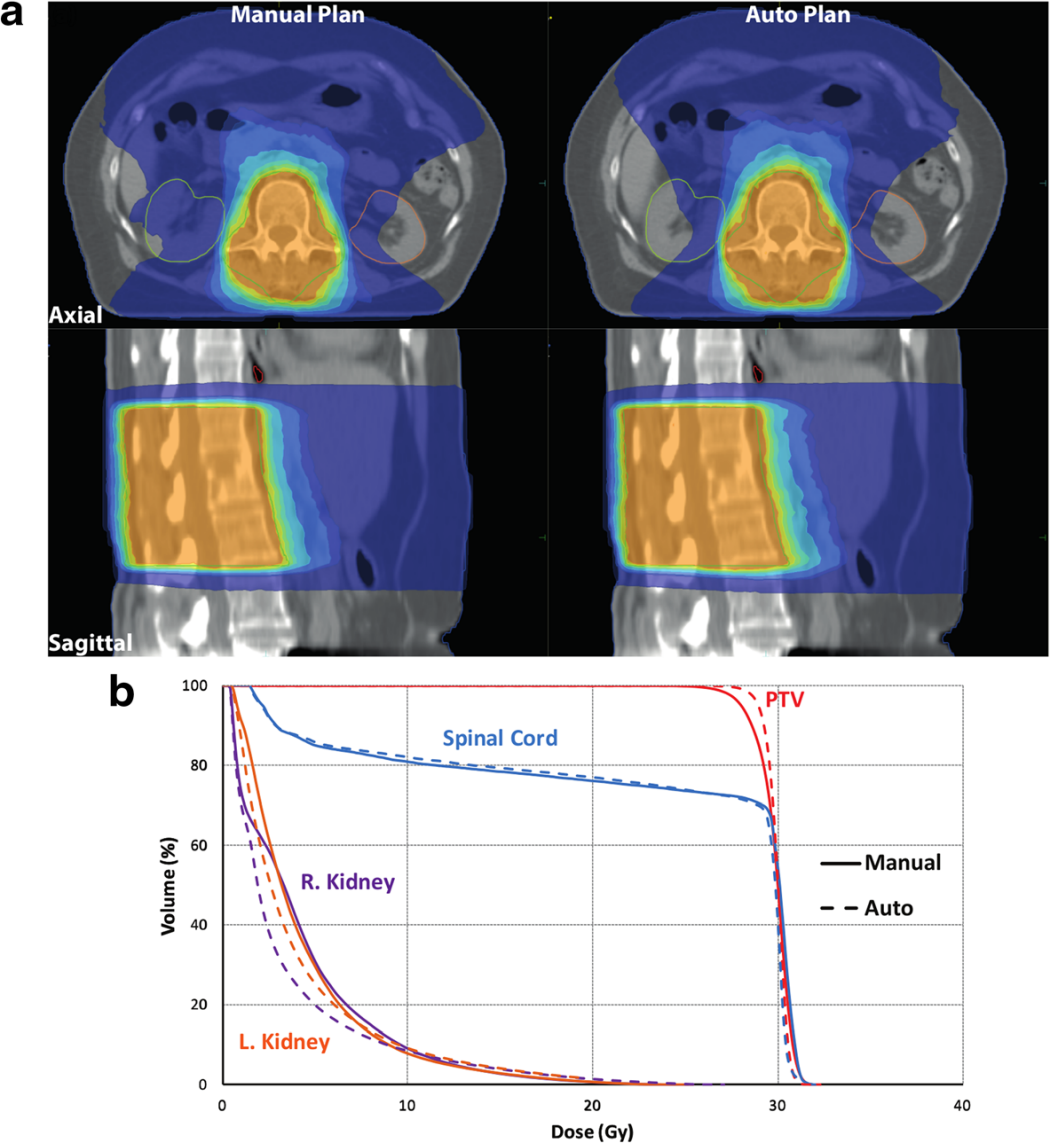
**a** Dose distribution of a treatment plan on thoracolumbar level (T12-L2). The patient had metastatic breast cancer; irradiation dose applied with this plan was 30 Gy in 10 fractions. Manual plan shown on the left and auto plan on the right. **b** Dose-volume histogram at the same thoracolumbar level, continuous line represents manual plan, and dotted line represents auto plan

**Fig. 2:**
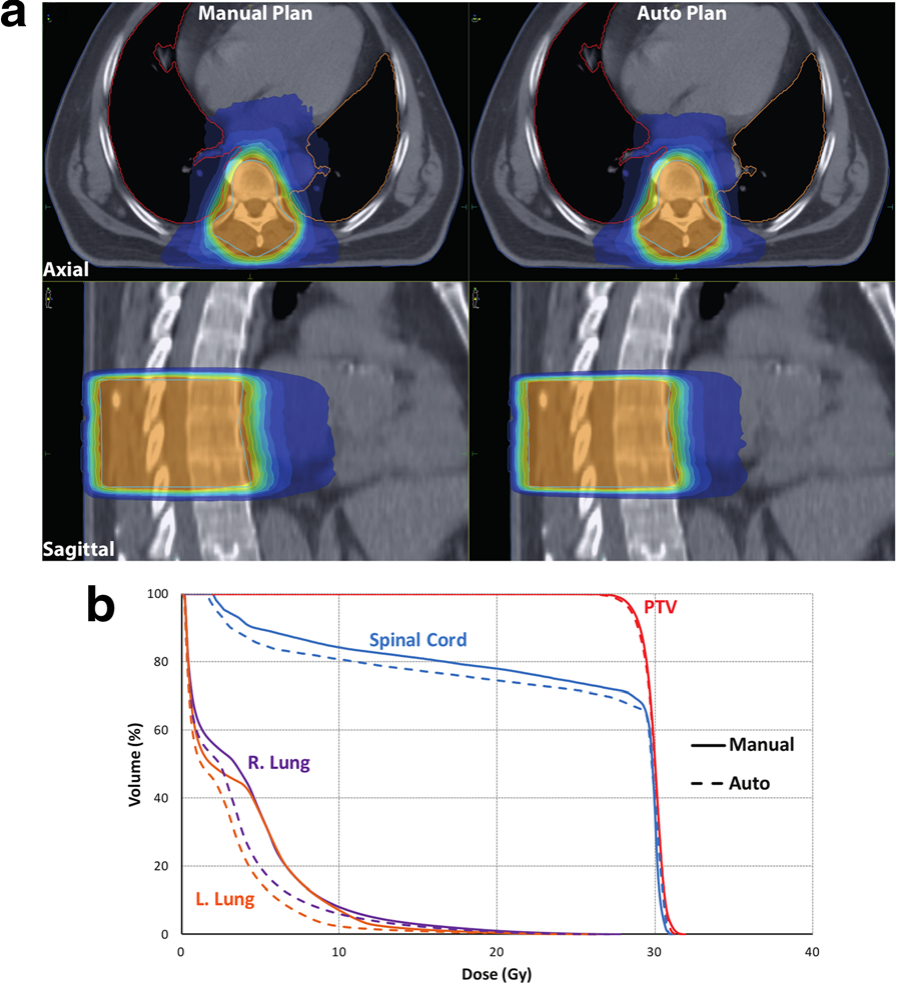
**a** Dose distribution of a treatment plan on thoracic level (T7-T9). The patient had metastatic prostate cancer; irradiation dose applied with this plan was 30 Gy in 10 fractions. Manual plan shown on the left and auto plan on the right. **b** Dose-volume histogram at the same thoracic level, continuous line represents manual plan, and dotted line represents auto plan

All automatically created plans were clinically deliverable and acceptable. Figures 1 and 2 show dose distributions, and dose-volume histogram comparisons for a kidney-level target, and a lung-level target, respectively.

### Target volumes

**Fig. 3:**
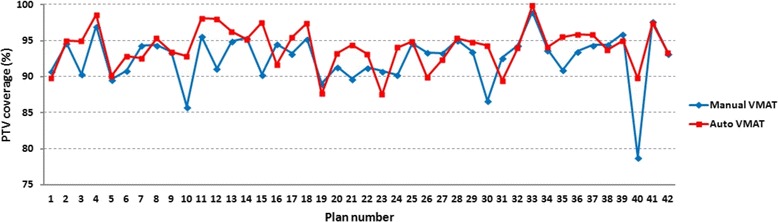
Volume receiving 95% of the prescribed dose, i.e., V_28.5Gy_ if prescribed dose was 30 Gy or V_38Gy_ if prescribed dose was 40 Gy

PTV coverage, defined as V_38Gy_ or V_28.5Gy_ was higher in auto plans when compared to manual plans when the whole patient population was analyzed, with an average difference of 1.47% (SD = 3.17%, *p* = 0.008, *n* = 42), see Fig. 3. When subgroups were analyzed, the difference was only significant in lung-level plans (*p* = 0.004, *n* = 20) but not at kidney levels (*p* = 0.110, *n* = 25). This pattern occurred irrespective whether 3 patients with both kidneys and lungs as OARs in one plan were added to the lung or to the kidney group or to both. Dose parameters were as follows: D_98%_ was numerically higher in auto plans when compared to manual plans but this was not statistically significant in the whole population (*p* = 0.479), nor in any subgroup (*p* > 0.05 for both lung and kidney levels). D_2%_ was significantly higher in manual plans over the whole patient population (*p* < 0.001), however the difference was clinically irrelevant (mean: 33.4 Gy vs. 33.2 Gy; median 31.1 Gy vs. 31.0 Gy). Subgroup analysis showed that D_2%_ was significantly lower in auto plans at kidney level (*p* = 0.003), however the difference was marginal and not clinically relevant. In lung-level plans, D_2%_ did not differ significantly between auto and manual plans.

HI as defined above was not significantly different between auto and manual plans in the whole group or in any subgroup (*p* > 0.05 for all comparisons). Considering the whole population, CI was slightly but statistically significant higher in auto plans than in manual plans: 1.014 ± 0.12 (1 SD) vs. 1.044 ± 0.11. For targets at the kidney level, the mean CIs for manual plans and auto plans were 0.99 ± 0.071 and 1.022 ± 0.067, respectively, and for lung-level targets 1.036 ± 0.157 and 1.065 ± 0.144. The differences between auto and manual plans can be explained by a slightly more pronounced underdosage in manual plans (minimum CI in manual plans: 0.88 [kidney level] and 0.81 [lung level]; minimum CI in auto plans: 0.92, and 0.95, respectively). C_diff_ did not differ significantly between auto and manual plans.

### Organs at risk

**Fig. 4:**
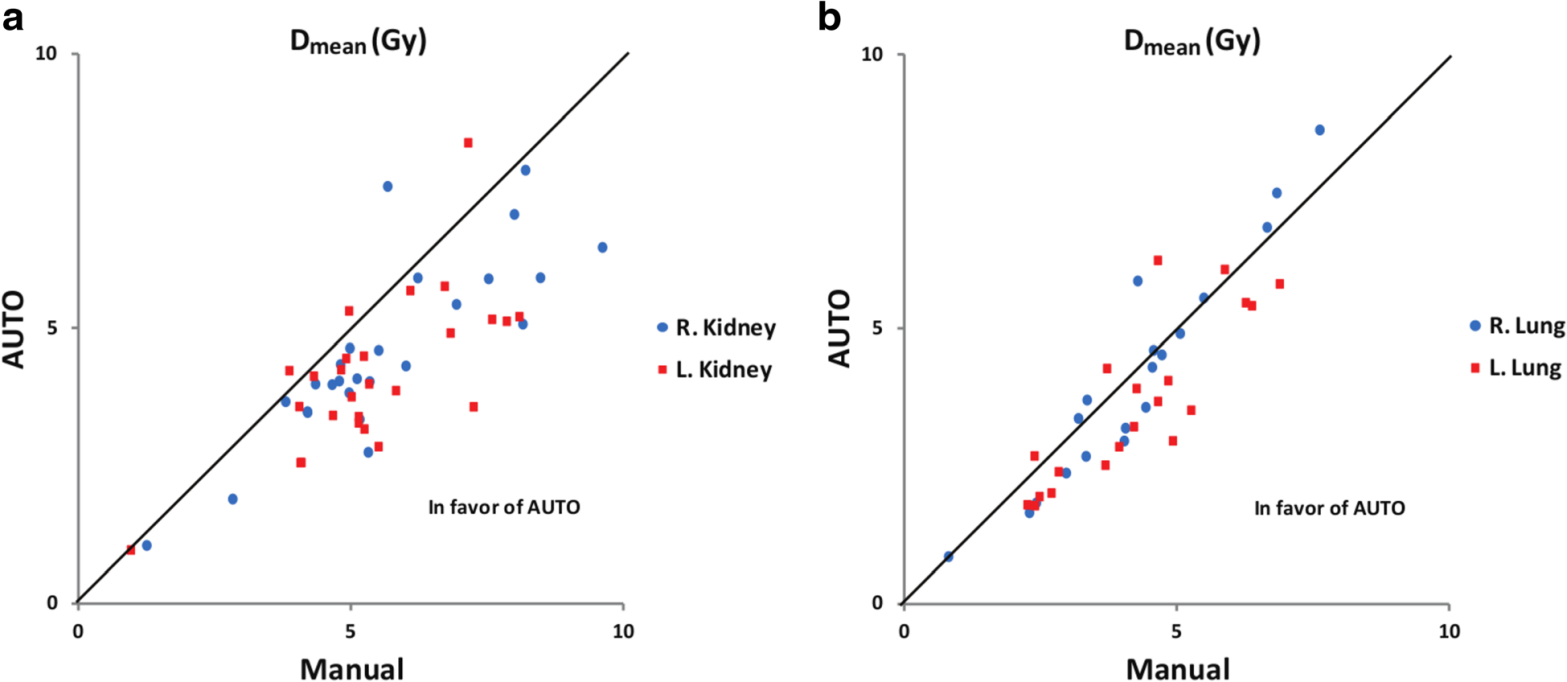
Comparison of mean dose to OARs. Each marker represents the mean dose in the manual plan vs. the auto plan to the kidneys (**a**) and to the lung (**b**). For data points right of the unity line, auto-planning yielded better sparing of OARs

In the pooled analysis of all plans, mean dose to OARs (kidneys and lungs pooled) was significantly lower in auto plans when compared to manual plans on both the right side (*p* = 0.001, *n* = 45, relative difference -10.9% [mean value auto vs. manual]) and on the left side (*p* < 0.001, *n* = 45, -19.5%). Subgroup analyses in patients with kidneys as OARs, including 3 patients with lungs and kidneys, showed that auto plans were associated with lower dose to both kidneys (*p* < 0.001, *n* = 25, -18.6% right kidney, and *p* < 0.001, *n* = 25, -23.1%, left kidney), see Fig. 4a for details. In lung-level plans, again including 3 plans in which both lungs and kidneys were included as OARs, only the left lung showed a significantly lower mean dose when auto-planning was used (*p* = 0.004, *n* = 20, -13.7%). Mean dose to the right lung did not differ significantly between manual and auto plans (*p* = 0.827, *n* = 20, -1.3%), see Fig. 4b for details. There was no significant difference between the maximum doses (D_max_) to the spinal cord between auto and manual groups in the whole population or in any subgroup. However, one patient in the auto group had a hotspot in the spinal cord (>110% of prescribed dose: 33.2 Gy, D_50%_: 30 Gy).

### Estimated treatment time and required monitor units

Auto plans required slightly more (estimated) treatment time in the whole population (*p* < 0.001, relative difference 7%) and in the subgroups of kidney-level (*p* < 0.001, relative difference 9.7%), and lung-level plans (*p* = 0.046, relative difference 3.6%) as no hard constraint was placed on treatment time. This was a consequence of auto plans requiring more monitor units in the whole population and in any subgroup (p-values and relative differences as follows: whole population, *p* < 0.001, 17.9%; kidney levels: *p* < 0.001, 15.4%; lung levels: *p* = 0.001, 20.6%). All target volume information, monitor units, OAR dose values, and treatment times for each patient are also shown in Additional file 1: Table S1.

## Discussion

Though highly conformal radiotherapy is not mandatory in the treatment of painful bone metastases [16], intensity modulation with static beams or VMAT allows to deliver a highly focused dose distribution in every clinical situation and may be beneficial for patients with oligometastatic disease [17] who require dose escalation or patients who had received prior courses of radiotherapy limiting doses to OARs in case of reirradiation [18, 19]. Highly conformal radiotherapy is, however, not always applied when appropriate [3] for its perceived resource intensity, both in personnel and planning time. Unexpectedly, the reason for not performing advanced radiotherapy techniques despite a clear indication seems to be a shortage of sufficiently trained personnel rather than machine shortage [3, 4, 20]. AlDuhaiby et al. described in a Canadian survey in 2012 that limitations in CT simulator or linear accelerator configuration impaired IMRT implementation in only 10% of cases [20]. The most relevant factors hindering IMRT implementation was the need to train existent treatment planners (50%) as well as the need to hire more planners (30%) [20]. Personal shortage is even more relevant in VMAT settings as these are more time consuming as compared to fixed field IMRT [21]; “increased planning time required for generating the VMAT plans” has been described by Rao et al. as the main disadvantage of VMAT [22]. Therefore, there is a need for tools that reduce planning time and ease the process of developing a radiotherapy plan without compromising plan quality.

Various auto-planning strategies are currently being developed [8], these include approaches for beam angle selection (e.g., [23]), and integrated beam weight optimization algorithms. The latter can be based on global optimization [24], or on sequential beam selection [25]. As discussed above, multicriterial optimization systems have been proposed [11, 12], that enable the user to select the plan out of different sets of beam angles and intensity profiles after optimization (*a posteriori* setting). Template based auto-planning solutions are already commercially available and have been applied to various target paradigms such as head and neck [26], breast [27] and esophageal targets [28].

Erasmus-iCycle plans, which provide a template-free approach have been successfully applied to head and neck [29], prostate [5], and cervical cancer [13] target volumes to create clinically deliverable plans.

Our results show that fully automated VMAT treatment planning with the Erasmus-iCycle/Monaco system for spinal metastases was non-inferior to conventional treatment planning by expert dosimetrists or medical physicists. Automatically generated plans with this system outperformed manual plans in terms of sparing of OARs in 3 of 4 predefined organs and also PTV coverage was favorable when compared to manual plans. We observed one hotspot in an iCycle plan in the spinal cord (33.2 Gy, D_50%_: 30 Gy). This problem can be addressed by modifying the wish list to include a maximum dose cost function on the spinal cord. In our analysis, however, we applied a quadratic overdose cost function which lead to maximum doses that were comparable to maximum doses observed in manual plans in all but this patient and allowed for more degrees of freedom regarding dose reduction to other OAR. If the maximum dose has to be strictly controlled, e.g., in case of reirradiation, a maximum dose cost function should be applied. Otherwise, the situation would be evaluated during the manual approval process which might prompt occasional replanning with a maximum dose cost function. Monitor units and estimated treatment time were higher in auto plans; however, this difference was not considered clinically relevant in the treated patient population and resulted in a negligible prolongation of treatment times. The difference in treatment times was expected as the aim of this study was to generate high-quality plans with clinically acceptable delivery times. Monitor units and delivery times could be reduced in the auto-plans if required. In contrast to a study in a more complex setting such as head and neck cancer [29], the observed dosimetric advantages of Erasmus-iCycle for treatment of spinal metastases were on average of low clinical relevance although advantages across the population were also observed.

External audits have shown that experienced treatment centers may yield superior IMRT plans [30]. Therefore, as postulated most recently by Fogliata et al. [28], quality improvements of automated planning vs. manual plan generation may even be more pronounced in target paradigms that are not frequently treated in a particular center.

## Conclusion

Our data add to the growing evidence [5, 13, 29] that automated treatment planning might be an alternative to manual planning, reducing the workload of medical phyicists and dosimetrists while maintaining or improving plan quality.

## Additional files

Additional file 1: ﻿Detailed comparison of auto- and manual planning results.﻿ (PDF 274 kb)
Additional file 2: Wish list for automatic plan generation. (DOCX 26 kb)

## Abbreviations

CI: Conformity index
CTV: Clinical target volume
ECOG: Eastern Cooperative Oncology Group
HI: Homogeneity index
IMRT: Intensity modulated radiation therapy
KPS: Karnofsky performance status
OAR: Organ at risk
PTV: Planning target volume
SBRT: Stereotactic body radiation therapy
TPS: Treatment planning system
VMAT: Volumetric modulated arc therapy

## References

[1] Tiwana MS, Barnes M, Kiraly A, Olson RA (2016). Utilization of palliative radiotherapy for bone metastases near end of life in a population-based cohort. BMC Palliat Care.

[2] Stieler F, Wolff D, Bauer L, Wertz HJ, Wenz F, Lohr F (2011). Reirradiation of spinal column metastases: comparison of several treatment techniques and dosimetric validation for the use of VMAT. Strahlenther Onkol.

[3] Mayles WP, Radiotherapy Development B (2010). Survey of the availability and use of advanced radiotherapy technology in the UK. Clin Oncol (R Coll Radiol).

[4] Shikama N, Tsujino K, Nakamura K, Ishikura S (2014). Survey of advanced radiation technologies used at designated cancer care hospitals in Japan. Jpn J Clin Oncol.

[5] Voet PW, Dirkx ML, Breedveld S, Al-Mamgani A, Incrocci L, Heijmen BJ (2014). Fully automated volumetric modulated arc therapy plan generation for prostate cancer patients. Int J Radiat Oncol Biol Phys.

[6] Breedveld S, Storchi PR, Keijzer M, Heemink AW, Heijmen BJ (2007). A novel approach to multi-criteria inverse planning for IMRT. Phys Med Biol.

[7] Breedveld S, Storchi PR, Heijmen BJ (2009). The equivalence of multi-criteria methods for radiotherapy plan optimization. Phys Med Biol.

[8] Breedveld S, Storchi PR, Voet PW, Heijmen BJ (2012). iCycle: Integrated, multicriterial beam angle, and profile optimization for generation of coplanar and noncoplanar IMRT plans. Med Phys.

[9] Rossi L, Breedveld S, Heijmen BJ, Voet PW, Lanconelli N, Aluwini S (2012). On the beam direction search space in computerized non-coplanar beam angle optimization for IMRT-prostate SBRT. Phys Med Biol.

[10] Voet PW, Breedveld S, Dirkx ML, Levendag PC, Heijmen BJ (2012). Integrated multicriterial optimization of beam angles and intensity profiles for coplanar and noncoplanar head and neck IMRT and implications for VMAT. Med Phys.

[11] Schreibmann E, Lahanas M, Xing L, Baltas D (2004). Multiobjective evolutionary optimization of the number of beams, their orientations and weights for intensity-modulated radiation therapy. Phys Med Biol.

[12] Craft D, Monz M (2010). Simultaneous navigation of multiple Pareto surfaces, with an application to multicriteria IMRT planning with multiple beam angle configurations. Med Phys.

[13] Sharfo AW, Voet PW, Breedveld S, Mens JW, Hoogeman MS, Heijmen BJ (2015). Comparison of VMAT and IMRT strategies for cervical cancer patients using automated planning. Radiother Oncol.

[14] Marks LB, Yorke ED, Jackson A, Ten Haken RK, Constine LS, Eisbruch A, Bentzen SM, Nam J, Deasy JO (2010). Use of normal tissue complication probability models in the clinic. Int J Radiat Oncol Biol Phys.

[15] Ohtakara K, Hayashi S, Hoshi H (2012). The relation between various conformity indices and the influence of the target coverage difference in prescription isodose surface on these values in intracranial stereotactic radiosurgery. Br J Radiol.

[16] Lutz S, Berk L, Chang E, Chow E, Hahn C, Hoskin P, Howell D, Konski A, Kachnic L, Lo S (2011). Palliative radiotherapy for bone metastases: an ASTRO evidence-based guideline. Int J Radiat Oncol Biol Phys.

[17] Corbin KS, Hellman S, Weichselbaum RR (2013). Extracranial oligometastases: a subset of metastases curable with stereotactic radiotherapy. J Clin Oncol.

[18] Kirkpatrick JP, van der Kogel AJ, Schultheiss TE (2010). Radiation dose-volume effects in the spinal cord. Int J Radiat Oncol Biol Phys.

[19] Damast S, Wright J, Bilsky M, Hsu M, Zhang Z, Lovelock M, Cox B, Zatcky J, Yamada Y (2011). Impact of dose on local failure rates after image-guided reirradiation of recurrent paraspinal metastases. Int J Radiat Oncol Biol Phys.

[20] AlDuhaiby EZ, Breen S, Bissonnette JP, Sharpe M, Mayhew L, Tyldesley S, Wilke DR, Hodgson DC (2012). A national survey of the availability of intensity-modulated radiation therapy and stereotactic radiosurgery in Canada. Radiat Oncol.

[21] Oliver M, Ansbacher W, Beckham WA (2009). Comparing planning time, delivery time and plan quality for IMRT, RapidArc and Tomotherapy. J Appl Clin Med Phys.

[22] Rao M, Yang W, Chen F, Sheng K, Ye J, Mehta V, Shepard D, Cao D (2010). Comparison of Elekta VMAT with helical tomotherapy and fixed field IMRT: plan quality, delivery efficiency and accuracy. Med Phys.

[23] Potrebko PS, McCurdy BM, Butler JB, El-Gubtan AS (2008). Improving intensity-modulated radiation therapy using the anatomic beam orientation optimization algorithm. Med Phys.

[24] Lee EK, Fox T, Crocker I (2006). Simultaneous beam geometry and intensity map optimization in intensity-modulated radiation therapy. Int J Radiat Oncol Biol Phys.

[25] de Pooter JA, Mendez Romero A, Wunderink W, Storchi PR, Heijmen BJ (2008). Automated non-coplanar beam direction optimization improves IMRT in SBRT of liver metastasis. Radiother Oncol.

[26] Hazell I, Bzdusek K, Kumar P, Hansen CR, Bertelsen A, Eriksen JG, Johansen J, Brink C (2016). Automatic planning of head and neck treatment plans. J Appl Clin Med Phys.

[27] Fogliata A, Nicolini G, Bourgier C, Clivio A, De Rose F, Fenoglietto P, Lobefalo F, Mancosu P, Tomatis S, Vanetti E (2015). Performance of a knowledge-based model for optimization of volumetric modulated Arc therapy plans for single and bilateral breast irradiation. PLoS One.

[28] Fogliata A, Nicolini G, Clivio A, Vanetti E, Laksar S, Tozzi A, Scorsetti M, Cozzi L (2015). A broad scope knowledge based model for optimization of VMAT in esophageal cancer: validation and assessment of plan quality among different treatment centers. Radiat Oncol.

[29] Voet PW, Dirkx ML, Breedveld S, Fransen D, Levendag PC, Heijmen BJ (2013). Toward fully automated multicriterial plan generation: a prospective clinical study. Int J Radiat Oncol Biol Phys.

[30] Nelms BE, Robinson G, Markham J, Velasco K, Boyd S, Narayan S, Wheeler J, Sobczak ML (2012). Variation in external beam treatment plan quality: an inter-institutional study of planners and planning systems. Pract Radiat Oncol.

