# Supplementary material for: Fully automated treatment planning of spinal metastases – A comparison to manual planning of Volumetric Modulated Arc Therapy for conventionally fractionated irradiation
Source: Radiat Oncol. 2017 Jan 31;12:33. doi: 10.1186/s13014-017-0767-2 (PMC5282882; doi:10.1186/s13014-017-0767-2)
Supplement: Additional file 1: — ﻿Detailed comparison of auto- and manual planning results.﻿ (PDF 274 kb) [file 13014_2017_767_MOESM1_ESM.pdf]

Plan Number	Patient number	Analyzed OAR	PTV location	Primary	PTV												R. Kidney		L. Kidney		R. Lung		L. Lung		Monitor Units (MU)		Treatment time		Spinal cord	
					Clinical	autoVMAT	Clinical	autoVMAT	Clinical	autoVMAT	Clinical	autoVMAT	Clinical	autoVMAT	Clinical	autoVMAT	Clinical	autoVMAT	Clinical	autoVMAT	Clinical	autoVMAT	Clinical	autoVMAT	Clinical	autoVMAT	Clinical	autoVMAT	Clinical	autoVMAT
					D98% (Gy)	D98% (Gy)	D2% (Gy)	D2% (Gy)	D50% (Gy)	D50% (Gy)	HI	HI	V28.5Gy or V38Gy(%)		CI	CI	Dmean (Gy)	Dmean (Gy)	Dmean (Gy)	Dmean (Gy)	Dmean (Gy)	Dmean (Gy)	Dmean (Gy)	Dmean (Gy)	# MU	# MU	t (sec)	t (sec)	Dmax (Gy)	Dmax (Gy)
1	1	Kidneys	T11-L5	Prostate	27.2	26.5	31.3	31.3	30.0	30.0	0.137	0.158	90.8	89.8	0.9894	0.9797	5.1	4.1	5.2	3.1					525	581	77	85	31.9	31.7
2	2	Kidneys	T12-L5	Prostate	27.6	26.9	31.0	30.9	30.0	30.0	0.112	0.135	94.7	95.0	1.0213	1.0381	5.0	4.6	5.0	3.7					708	791	194	193	31.7	31.9
3	3	Kidneys	T11-L2	Breast	27.0	27.8	31.1	31.0	30.0	30.0	0.139	0.105	90.3	94.9	0.9338	1.0149	5.1	3.3	4.9	4.4					734	805	163	176	31.5	31.4
5	4	Kidneys	T10-L6	Multiple Myeloma	36.4	35.9	41.7	41.3	40.0	40.0	0.133	0.136	89.6	90.1	0.9515	1.0138	8.0	7.1	7.8	5.1					473	517	156	173	43.3	42.0
6	5	Kidneys	T10-S3	Breast	35.9	35.1	41.5	41.2	40.0	40.0	0.138	0.155	90.9	92.9	0.9503	1.0080	6.9	5.4	6.8	4.9					424	446	141	151	41.8	41.9
7	6	Kidneys	L2-L5	Breast	36.8	35.7	41.4	41.3	40.0	40.0	0.115	0.141	94.4	92.6	0.9956	0.9703	8.2	7.9	7.6	5.1					394	474	152	172	42.2	42.4
9	7	Kidneys	L1-S5	Prostate	27.5	24.8	31.0	30.9	30.0	30.0	0.116	0.206	93.4	93.4	0.9840	1.0166	5.3	2.7	5.5	2.8					581	599	186	239	28.9	30.7
11	9	Kidneys	T11-L1	Prostate	28.1	28.5	30.9	30.9	30.0	30.0	0.095	0.078	95.6	98.2	1.0428	1.0979	4.8	4.0	4.1	2.5					639	753	164	170	31.4	31.1
12	10	Kidneys	T12-L2	Breast	27.3	28.5	31.3	30.9	30.0	30.0	0.132	0.079	91.1	98.0	0.9684	1.1071	4.2	3.5	4.3	4.1					565	668	163	168	32.0	31.7
14	11	Kidneys	T11-L2	NSCLC	28.1	27.9	31.2	31.0	30.0	30.0	0.104	0.104	95.4	95.2	1.0434	1.0402	6.0	4.3	5.8	3.8					615	753	178	178	31.5	31.4
15	12	Kidneys	T10-L4	Multiple Myeloma	27.2	28.4	31.1	30.8	30.0	30.0	0.132	0.082	90.3	97.5	0.9234	1.0258	4.6	4.0	4.7	3.4					585	788	126	134	31.5	32.0
16	13	Kidneys	T12-L2	Breast	27.8	26.3	31.0	31.2	30.0	30.0	0.108	0.163	94.5	91.7	1.0277	0.9904	8.4	5.9	7.2	3.5					547	720	165	171	31.4	31.2
17	14	Kidneys	L1-L5	Oesophageal	27.0	27.3	31.5	30.9	30.0	30.0	0.150	0.119	93.3	95.5	1.0085	1.0532	5.7	7.6	5.2	4.5					776	861	180	202	32	31.4
19	15	Kidneys	T12-L5	Prostate	35.3	29.8	41.9	41.4	40.0	40.0	0.165	0.291	89.1	87.6	0.9171	0.9552	8.1	5.1	8.1	5.2					498	557	163	183	41.9	43.7
21	16	Kidneys	T12-L3	H & N SCC	26.6	26.2	31.2	30.9	30.0	30.0	0.154	0.157	89.7	94.4	0.9828	1.0792	6.2	5.9	6.1	5.7					702	763	171	179	31.3	31.1
22	17	Kidneys	L1	Breast	36.4	36.6	41.3	41.5	40.0	40.0	0.123	0.123	91.3	93.2	0.9743	1.0048	4.3	4.0	4.9	5.3					363	435	123	122	42.1	42.3
23	18	Kidneys	T12-S1	Prostate	26.5	22.0	31.2	31.1	30.0	30.0	0.156	0.302	90.8	87.5	0.9488	0.9206	5.3	4.0	5.1	3.4					742	805	240	259	31.6	31.4
26	20	Kidneys	T8-S5 / pelvis	Breast	36.6	32.0	41.8	41.2	40.0	40.0	0.129	0.231	93.4	89.9	1.0294	1.0073	9.6	6.5	6.7	5.7					652	702	267	312	42.5	41.7
28	21	Kidneys	T9-L1	Adenocarcinoma CUP	27.5	27.7	31.0	30.9	30.0	30.0	0.116	0.108	95.1	95.3	1.0426	1.0268	3.8	3.6	3.9	4.2					778	842	137	212	31.2	31.2
30	23	Kidneys	T12-L1	Breast	26.9	27.8	31.0	31.0	30.0	30.0	0.135	0.108	86.7	94.3	0.8799	0.9706	4.9	3.8	5.1	3.3					584	808	124	132	31.1	31
31	24	Kidneys	T8-L3	SCLC	27.2	25.5	31.0	31.3	30.0	30.0	0.129	0.194	92.7	89.5	1.0012	0.9497	5.5	4.6	5.3	4.0					787	905	133	147	31.5	33.2
33	25	Kidneys	L3	Gastric Cancer	28.8	29.2	30.8	30.8	30.0	30.0	0.066	0.053	99.0	99.9	1.2480	1.2620	1.2	1.0	1.0	0.9					452	614	156	160	31.2	31.1
10	8	Kidneys and Lungs	T10-L3	Urothelial	35.7	36.9	41.9	41.4	40.0	40.0	0.153	0.111	85.8	92.9	0.8796	0.9771	7.5	5.9	7.1	8.4	3.2	3.4	2.4	2.7	475	519	159	181	42.4	42.2
25	19	Kidney and Lung	T11-L1	NSCLC	27.8	27.5	31.0	30.9	30.0	30.0	0.106	0.113	94.7	94.9	1.0256	1.0047	2.8	1.9	4.0	3.6	0.8	0.8	2.2	1.8	537	711	168	165	31.7	31
29	22	Kidneys and Lungs	T7-L1	Breast	27.0	26.6	31.9	31.0	30.0	30.0	0.164	0.146	93.5	94.8	0.9896	1.0346	4.8	4.3	4.8	4.2	5.0	4.9	4.2	3.9	1113	1185	170	184	30.7	31.5
34	25	Lungs	T9-T11	Gastric	27.6	27.2	30.9	31.0	30.0	30.0	0.110	0.128	93.7	94.1	1.0268	1.0318					3.3	2.7	2.8	2.4	472	628	162	158	31	31.5
35	25	Lungs	T3-T6	Gastric	27.2	27.9	31.1	30.9	30.0	30.0	0.130	0.100	90.9	95.6	0.9823	1.0620					3.3	3.7	3.7	4.3	549	693	159	181	31.7	30.9
36	26	Lungs	T5-T10	Breast	27.8	28.1	31.1	30.9	30.0	30.0	0.110	0.094	93.5	95.9	1.0047	1.0524					6.6	6.9	6.2	5.5	442	462	87	93	31.6	31.6
37	27	Lungs	T3-T6	Prostate	27.8	27.9	30.9	31.1	30.0	30.0	0.102	0.107	94.4	95.8	1.0050	1.0461					4.4	3.6	4.6	3.7	434	617	84	101	31.6	31.5
38	28	Lungs	T6-T11	Prostate	27.9	27.8	31.2	31.0	30.0	30.0	0.107	0.107	94.5	93.8	1.0262	0.9875					5.5	5.6	5.2	3.5	443	551	81	104	31.8	31.0
39	29	Lungs	T7-T9	Prostate	28.1	27.9	31.1	31.0	30.0	30.0	0.100	0.104	96.0	95.0	1.0582	1.0347					4.0	3.2	3.7	2.5	530	617	162	150	31.2	31.0
40	30	Lungs	T3-T8	NSCLC (Adeno)	22.6	25.6	32.4	31.0	30.0	30.0	0.325	0.180	78.8	89.8	0.8074	0.9525					4.3	5.9	4.6	6.2	648	621	155	179	32.8	31.3
41	31	Lungs	C6-T4	Breast	28.4	28.3	30.9	30.8	30.0	30.0	0.082	0.083	97.7	97.4	1.1740	1.1120					2.4	1.8	2.5	1.9	652	722	193	209	31.1	31.1
42	32	Lungs	T2-T4	Multiple Myeloma	27.7	27.3	31.1	31.0	30.0	30.0	0.111	0.121	93.2	93.3	0.9727	0.9816					3.0	2.4	2.7	2.0	490	810	122	137	31.8	31.4
4	3	Lungs	T6-T8	Breast	28.3	28.7	30.9	30.8	30.0	30.0	0.088	0.073	97.0	98.6	1.0576	1.0950					4.5	4.3	3.9	2.9	595	640	169	164	31.4	31.3
8	6	Lungs	T6-T11	Breast	37.0	37.0	41.4	41.1	40.0	40.0	0.112	0.103	94.4	95.4	1.0027	1.0291					7.6	8.6	6.9	5.8	354	432	159	172	41.8	42.7
13	10	Lungs	C7-T5	Breast	27.9	28.0	31.0	30.9	30.0	30.0	0.101	0.095	94.9	96.3	1.0009	1.0787					4.7	4.5	4.8	4.1	457	610	161	165	31.1	31.3
18	14	Lungs	T7-T10	Oesophageal	28.0	28.4	31.1	30.9	30.0	30.0	0.103	0.084	95.3	97.5	1.0122	1.0498					4.6	4.6	4.9	3.0	463	691	202	160	31	31.1
20	15	Lungs	T3-T8	Prostate	35.8	36.4	41.4	41.4	40.0	40.0	0.140	0.124	91.4	93.3	1.6230	1.6526					6.8	7.5	6.4	5.4	373	448	163	148	41.5	41.6
24	18	Lungs	T2-T9	Prostate	26.4	27.3	31.3	31.0	30.0	30.0	0.164	0.126	90.3	94.1	0.9483	1.0087					9.3	11.8	5.9	6.1	1115	1009	269	285	32.1	31.7
27	20	Lungs	T3	Breast	27.7	27.1	30.9	31.1	30.0	30.0	0.106	0.132	93.3	92.4	1.0955	1.0665					2.3	1.6	2.4	1.8	452	689	153	159	31.4	30.9
32	24	Lungs	T4-T5	SCLC	27.9	27.6	30.9	31.0	30.0	30.0	0.100	0.112	94.3	94.0	1.0253	1.0379					4.0	3.0	4.2	3.2	572	806	172	168	31.2	31.3
