# Supplementary material for: Fully automated treatment planning of spinal metastases – A comparison to manual planning of Volumetric Modulated Arc Therapy for conventionally fractionated irradiation
Source: Radiat Oncol. 2017 Jan 31;12:33. doi: 10.1186/s13014-017-0767-2 (PMC5282882; doi:10.1186/s13014-017-0767-2)
Supplement: Additional file 2: — Wish list for automatic plan generation. (DOCX 26 kb) [file 13014_2017_767_MOESM2_ESM.docx]

Supplementary Table 2. Wish-list for automatic plan generation.

| Constraints |  |  |  |  |
| --- | --- | --- | --- | --- |
|  | **Volume** | **Type** | **Limit** |  |
|  | PTV | Max | 105% of *D^p^* |  |
|  | Unspecified Tissues | Max | 105% of *D^p^* |  |
| Objectives |  |  |  |  |
| **Priority** | **Volume** | **Type** | **Goal** | **Parameters** |
| **1** | PTV | ↓ LTCP | 0.5 | *D^p^* = 30(40) Gy, α = 0.75 |
|  |  |  |  |  |
| **2** | PTV Shell 5 mm | ↓ Max | 80% of *D^p^* |  |
|  | PTV Shell 20 mm | ↓ Max | 50% of *D^p^* |  |
|  | Skin Ring 30 mm | ↓ Max | 25% of *D^p^* |  |
|  | Right Kidney | ↓ Mean | 15% of *D^p^* |  |
|  | Left Kidney | ↓ Mean | 15% of *D^p^* |  |
|  | Right Lung | ↓ Mean | 15% of *D^p^* |  |
|  | Left Lung | ↓ Mean | 15% of *D^p^* |  |
|  | PTV Shell 40 mm | ↓ Max | 10% of *D^p^* |  |
|  |  |  |  |  |
| **3** | PTV Shell 50 mm | ↓ Max | 10% of *D^p^* |  |

*Abbreviations*: *D^p^* = prescribed dose, LTCP = Logarithmic Tumor Control Probability, α = cell sensitivity.
